# Supplementary material for: Atomic fluctuations lifting the energy degeneracy in Si/SiGe quantum dots
Source: Nat Commun. 2022 Dec 13;13:7730. doi: 10.1038/s41467-022-35458-0 (PMC9747794; doi:10.1038/s41467-022-35458-0)
Supplement: Supplementary file 1 — Supplementary Information [file 41467_2022_35458_MOESM1_ESM.pdf]

Supplementary Information: Atomic fluctuations lifting the energy degeneracy in Si/SiGe quantum dots

Brian Paquelet Wuetz,[1,*] Merritt P. Losert,[2,*] Sebastian Koelling,[3,*] Lucas E.A. Stehouwer,[1] Anne-Marije J. Zwerver,[1] Stephan G.J. Philips,[1] Mateusz T. Mądzik,[1] Xiao Xue,[1] Guoji Zheng,[1] Mario Lodari,[1] Sergey V. Amitonov,[1] Nodar Samkharadze,[4] Amir Sammak,[4] Lieven M.K. Vandersypen,[1] Rajib Rahman,[5] Susan N. Coppersmith,[5] Oussama Moutanabbir,[3] Mark Friesen,[2] and Giordano Scappucci[1,†]

[1] *QuTech and Kavli Institute of Nanoscience, Delft University of Technology, PO Box 5046, 2600 GA Delft, The Netherlands*
[2] *University of Wisconsin-Madison, Madison, WI 53706 USA*
[3] *Department of Engineering Physics, École Polytechnique de Montréal, Montréal, Case Postale 6079, Succursale Centre-Ville, Montréal, Québec, Canada H3C 3A7*
[4] *QuTech and Netherlands Organisation for Applied Scientific Research (TNO), Delft, The Netherlands.*
[5] *University of New South Wales, Sydney, Australia*

(Dated: November 23, 2022)

CONTENTS

1. Electrical characterization 2
   a. Magnetotransport characterisation of Hall-bar shaped heterostructure field effect transistors 2
   b. Singlet-triplet energy splitting in quantum dots 5

2. Material characterization 9
   a. Atom Probe Tomography analysis of interfaces 9
   b. Extraction of the cubes and Voronoi tessellation 9
   c. Construction of the interface 10
   d. Generating model data 13
   e. Atomic steps, Quantum well width, and bottom interfaces 15
   f. SIMS and crosshatch pattern 16

3. Theoretical model 17
   a. Tight-binding model 17
   b. Comparison with NEMO-3D 17
   c. Statistical distribution of intervalley couplings 19
   d. Statistical distribution of valley splittings 21
   e. Effects of interface width and QW Ge concentration on average valley splitting 21
   f. Effect of vertical electric field on average valley splitting 21

References 24

* These authors contributed equally
† g.scappucci@tudelft.nl

1. ELECTRICAL CHARACTERIZATION

a. Magnetotransport characterisation of Hall-bar shaped heterostructure field effect transistors

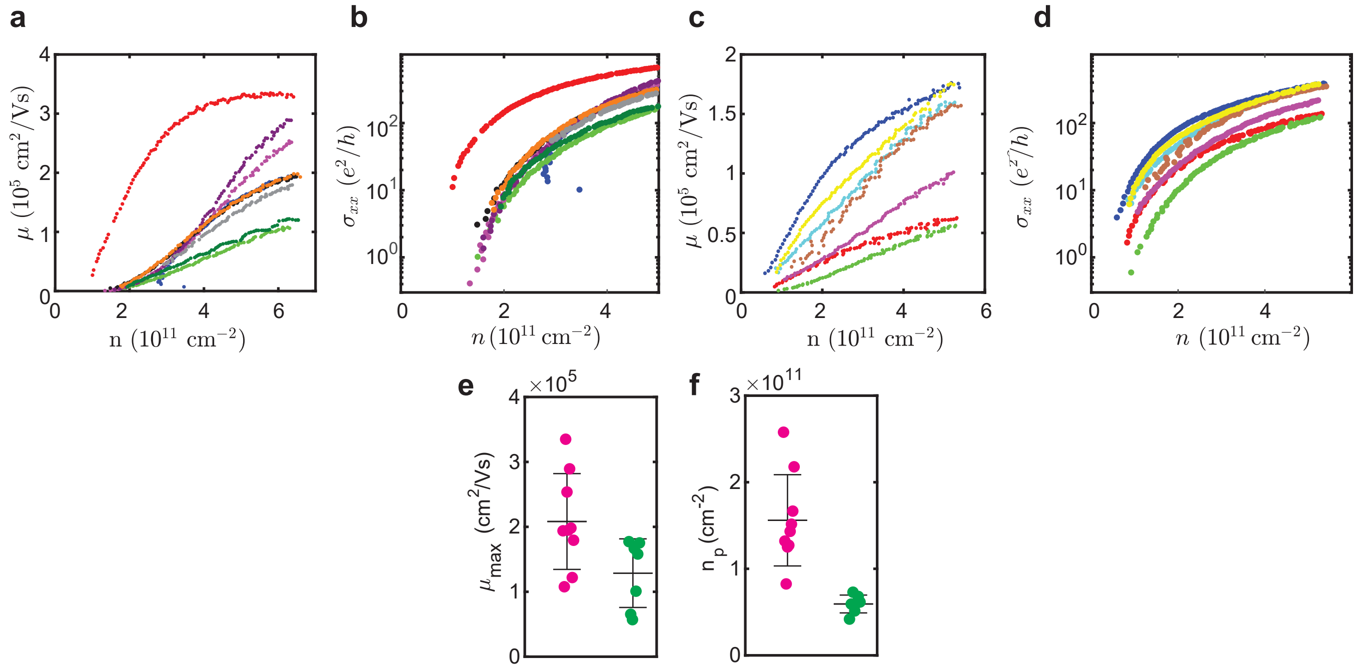

Supplementary Figure 1. **a**,**b**. Mobility μ and conductivity σ_{xx} as a function of Hall density n measured for quantum well A. **c**, **d** Mobility μ and conductivity σ_{xx} as a function of Hall density n measured for quantum well B. **e** Maximum mobility μ_{max} for quantum well A (magenta) and quantum well B (green) extracted from **a** and **c**. Black crosses are the mean and standard deviation. For quantum well A we find $\overline{\mu_{max}} = 129.000 \pm 53.000$ cm^2/Vs and for quantum well B we find $\overline{\mu_{max}} = 208.000 \pm 74.000$ cm^2/Vs. **f** Percolation density n_p for quantum well A (magenta) and quantum well B (green) extracted by fitting the conductivity-density curves in **b** and **d** to the relationship $\sigma_{xx} \propto (n - n_p)^{1.31}$[1]. Since this percolation theory is valid only at low densities, for each sample we chose a fitting range that goes from the lowest measured density n_{min} to a density $n_{max,fit}$ that yields the best fitting results. For the devices from quantum well A in **b** we have $n_{max,fit} = 3.2\times10^{11}$ cm^{-2}, 2.2×10^{11} cm^{-2}, 2×10^{11} cm^{-2}, 2×10^{11} cm^{-2}, 2.2×10^{11} cm^{-2}, 2.2×10^{11} cm^{-2}, 2.5×10^{11} cm^{-2}, 4×10^{11} cm^{-2}, 5.8×10^{11} cm^{-2}. For the devices from quantum well B in **d** we have $n_{max,fit} = 1.35\times10^{11}$ cm^{-2}, 1.35×10^{11} cm^{-2}, 1.6×10^{11} cm^{-2}, 1.6×10^{11} cm^{-2}, 1.6×10^{11} cm^{-2}, 1.8×10^{11} cm^{-2}, 1.35×10^{11} cm^{-2} Black crosses are the mean and standard deviation of the percolation density. For quantum well A we find $\overline{n_p} = 1.56 \pm 0.53\times10^{11}$ cm^{-2} and for quantum well B we find $\overline{n_p} = 0.59 \pm 0.1\times10^{11}$ cm^{-2}.

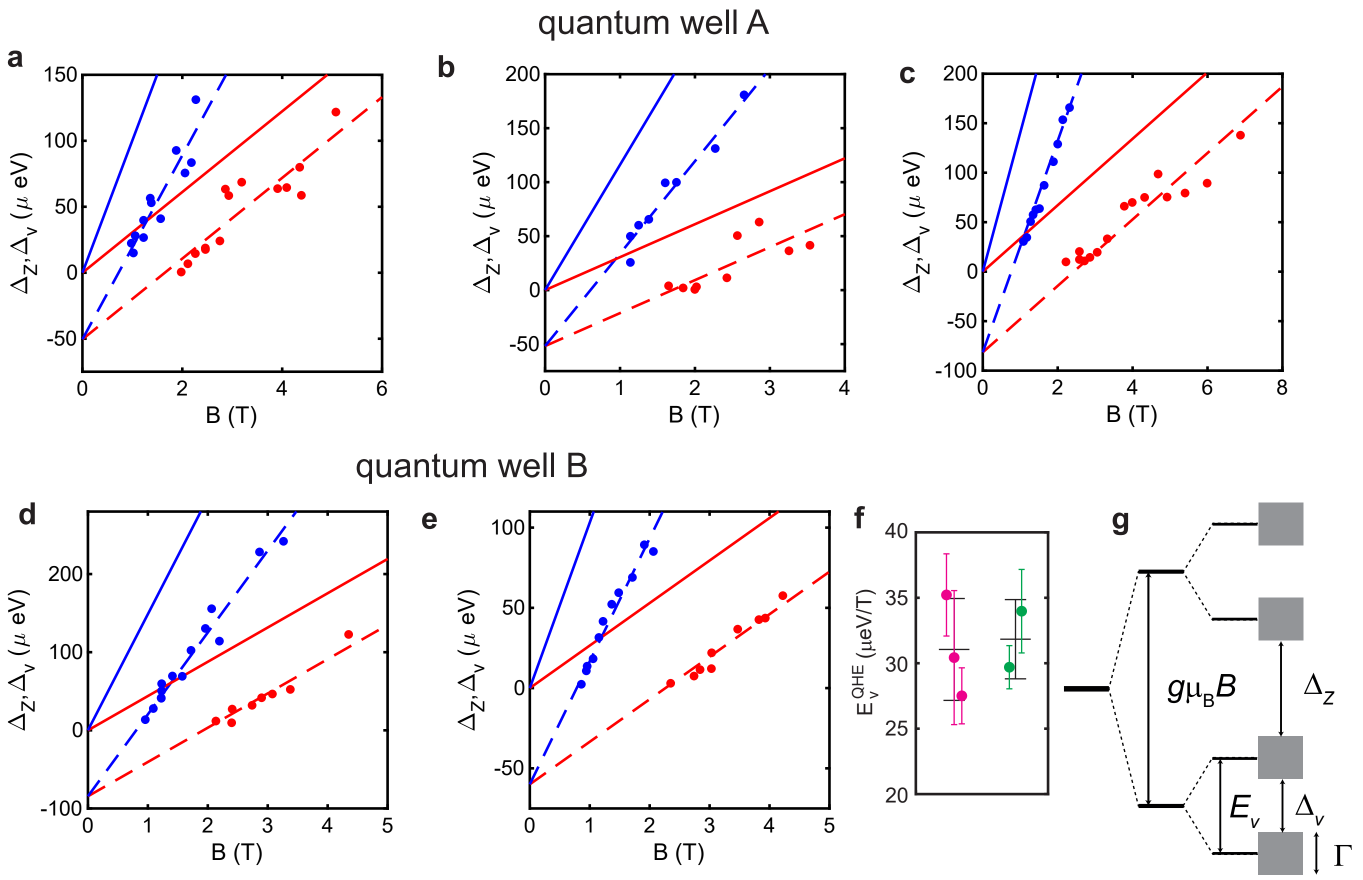

Supplementary Figure 2. **a**-**c** Activation energy measurements of the valley gap Δ_v (red circles) and Zeeman gap Δ_Z (blue circles) as a function of the magnetic field B for three different devices from quantum well A. Δ_v is measured at the $2n-1$ quantum Hall filling factors and Δ_Z is measured at the $4n-2$ filling factors. We follow the same methodology as in Ref. [2]. The blue and red dashed lines are theoretical fits to the experimental data using the equations $\Delta_Z = g^*\mu_B B - c_B B - \Gamma$ and $\Delta_v = c_B B - \Gamma$, where g^* is the effective Landé-g-factor, μ_B is the Bohr magneton, c_B is the proportionality factor of the valley splitting with B, and Γ is the Landau level broadening induced by disorder. We obtain $c_B = 30.64 \pm 3.14$ µeV/T, 30.43 ± 5.12 µeV/T, 32.46 ± 2.14 µeV/T, and $g^* = 1.74 \pm 0.16,\ 2 \pm 0.21,\ 2.36 \pm 0.12$ respectively. The blue and red solid lines correspond to the estimated Zeeman and valley energy gaps, respectively. **d**, **e** Activation energy measurements and fits of the valley gap and Zeeman gap as in **a**-**c** for two devices from quantum well B. We obtain $c_B = 26.28 \pm 1.65$ µeV/T, 43.15 ± 3.19 µeV/T, and $g^* = 1.77 \pm 0.13,\ 2.54 \pm 0.17$ respectively. **f** Rate of increase of valley splitting with magnetic field E_V^{QHE} for quantum well A (magenta) and quantum well B (green) extracted from the fitting analysis of **a**-**e**. We calculate E_V^{QHE} by setting $E_V^{QHE} = c_B g/g^*$, thereby scaling c_B with a coefficient g/g^* that normalizes the fitted g^* to the value $g = 2$ in silicon. This normalization is a way to take into account the modest electron-electron interaction present in different devices, allowing for a comparison across different quantum wells. Black crosses are the mean and standard deviation of E_V^{QHE}. For quantum well A we find $\overline{E_V^{QHE}} = 31.1 \pm 3.9$ µeV/T and for quantum well B we find $\overline{E_V^{QHE}} = 31.8 \pm 3$ µeV/T. **g**, Schematic drawing of a Landau level split into Zeeman and valley energy levels, showing all relevant energy separations. Shaded areas represent the single-particle Landau level broadening Γ due to disorder [2].

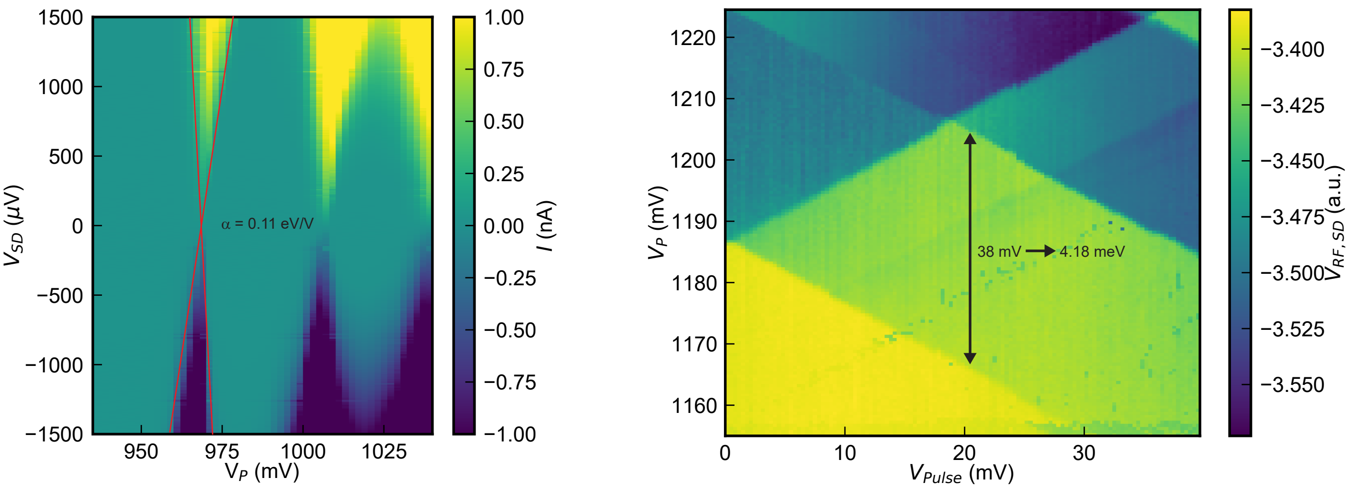

Supplementary Figure 3. **A** Coulomb blockade measurements of QD1, device 5 (see Table S3). The current through the QD is monitored while scanning the gate voltage and the bias voltage applied between the source and the drain, resulting in Coulomb diamonds. From the leftmost Coulomb diamond (indicated by the red lines) we extract a leverarm $\alpha = 0.11$ eV/V using the method described in the supplementary information of Ref. [3]. **b** Pulsed gate spectroscopy for the same quantum dot. The time-averaged RF reflectometry signal/sensing dot response is plotted as a function of the dc gate voltage V_P and the square pulse amplitude V_{pulse} with a pulse frequency of 25 kHz, both applied to the same gate. The arrow indicates the orbital splitting, which we extract as $E_{orb} = \alpha V_{orb} = 4.18$ meV, consitent with other values reported in literature [4–6].

b. Singlet-triplet energy splitting in quantum dots

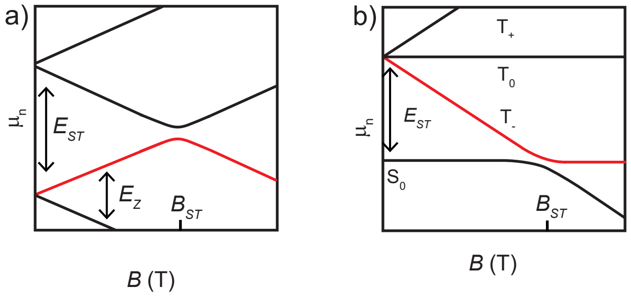

Supplementary Figure 4. **a**, Energy evolution of the ground state and first excited state in a single quantum dot as a function of the magnetic field. The red line shows the expected spin filling for the charge transition $N = 1 \rightarrow 2$. At $B = B_{ST}$ the typical kink can be observed, where the Zeeman energy E_Z is equal to the singlet-triplet splitting energy E_{ST}. **b**, Energy evolution of the four lowest lying energy states in a double quantum dot as a function of the magnetic field with fixed electron number $N = 2$. The red line represents the T_- energy state measured along the (1,1) $\rightarrow$(2,0) transition. At $B = B_{ST}$ the singlet state S_0 and the triplet state T_- are equal in energy, resulting in an anticrossing.

The singlet-triplet energy splitting is computed according to the configurations in Fig. 4. In the configuration in Fig. 4a the red line can be fitted to compute E_{ST} with the formula[7]:

$$V_P = \frac{1}{\alpha\beta_e} \ln \frac{e^{\frac{1}{2}\kappa B + \beta_e E_{ST}}(e^{\kappa B} + 1)}{e^{\kappa B} + e^{2\kappa B} + e^{\kappa B + \beta_e E_{ST}} + 1}, \tag{1}$$

where α is the lever arm converting gate voltage to energy, V_P is the gate voltage, $\kappa = g\mu_B\beta_e$ where $\beta_e = 1/k_B T_e$, g is the Lande-g-factor in silicon, μ_B is the Bohr magneton, B is the magnetic field, k_B is Boltzmann's constant, and T_e is the electron temperature[7].

In the configuration in Fig. 4b the Hamiltonian of the T$-$ state is given by:

$$\hat{H} = \begin{pmatrix} E_{S0} & t_c \\ t_c & E_{T-} \end{pmatrix} \tag{2}$$

where E_{S0} is the energy evolution of the singlet state, E_{T-} is the energy evolution of the triplet minus-state, and the off-diagonal element t_c is the tunnel coupling between the (1,1)-state and the the (2,0)-state in the double quantum dot. Diagonalization of the Hamiltonian yields:

$$\mu_n(T-) = \frac{1}{2}(E_{S0} + E_{T-} + \sqrt{(E_{S0} - E_{T-})^2 + 4t_c^2}\ ) \tag{3}$$

To fit the red line from Fig. 4b we use $E_{S0} = 0$ and $E_{T_-} = \alpha(g\mu_B B + E_{ST})$, where α is the lever arm, g is the single particle g-factor, B is the magnetic field, and E_{ST} is the singlet-triplet splitting.

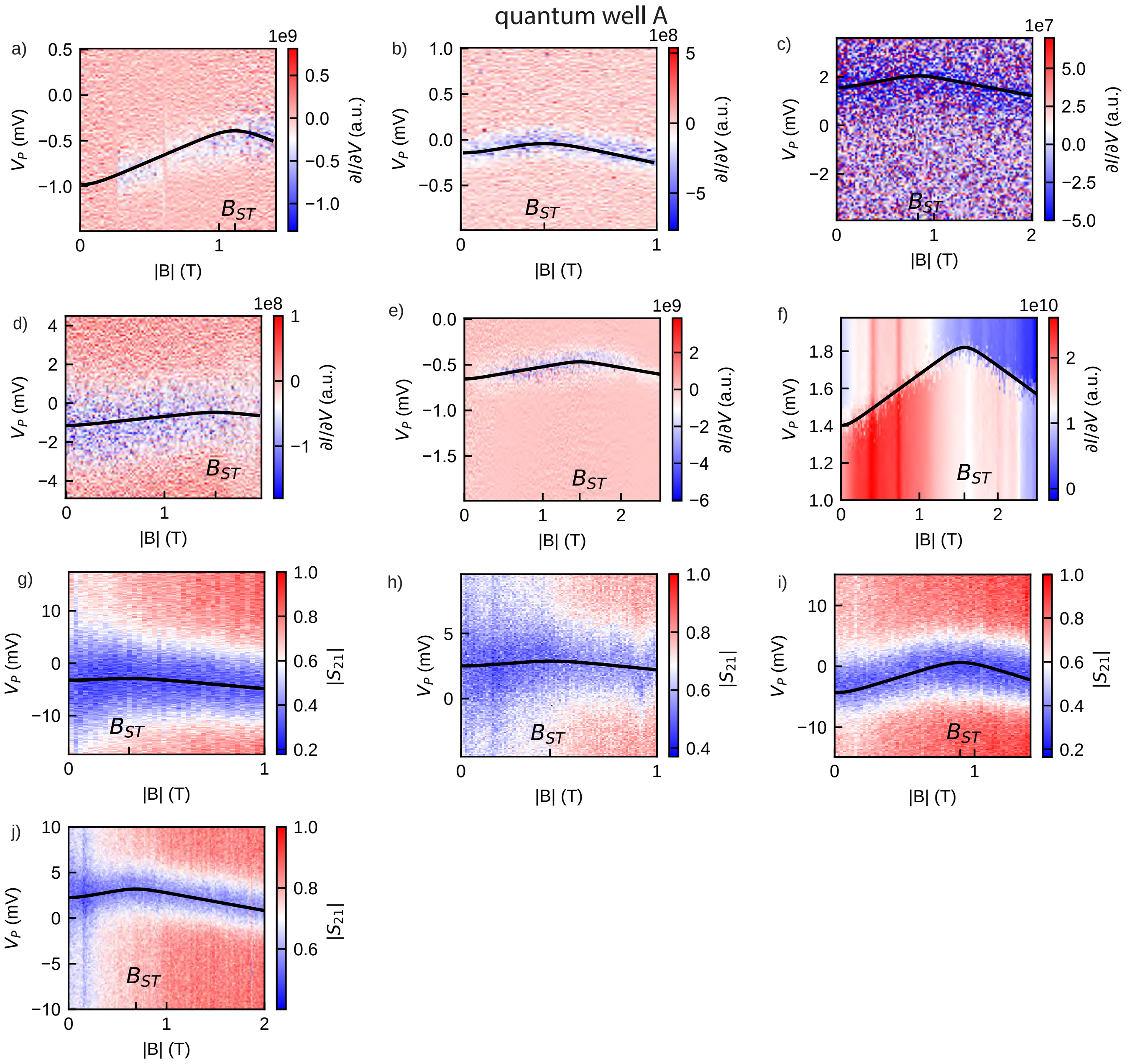

Supplementary Figure 5. Magnetospectroscopy of quantum dots fabricated on quantum well A. V_P is the gate voltage applied to the plunger gate forming the quantum dot. For clarity, we subtract from V_P in panels a) - j) an offset that depends is on the quantum dot being measured. a) - f) Magnetospectroscopy data measured along the $N = 1 \rightarrow 2$ transition of five different quantum dots on three different samples in quantum well A. The signal is measured by monitoring the derivative of the current through a nearby charge sensor. a), A charge fluctuation occurred during the measurement and to optimize the fitting routine, we shifted the data in the range 0.3-0.6 T upwards by 1 mV. a) - f), Due to low tunnel rates, for each gate Voltage sweep at the different magnetic fields, we determine the points with the highest derivative of the current $\frac{\partial I}{\partial V}$ through the charge sensor as the $N = 1 \rightarrow 2$ charge transition. We then use these points as the input of eq. 1. With this equation we can fit the charge transition as a function of the magnetic field (black curve). g) - j) Magnetospectroscopy data measured along the $N = 1 \rightarrow 2$ transition of four different quantum dots on two different samples in quantum well A. The quantum dot is probed via gate-based sensing using an on-chip superconducting resonator in these measurements [8]. The magnitude of the transmitted microwave signal S_{21} through a feed line that is capacitively coupled to the resonator is plotted here. For each gate Voltage sweep at the different magnetic fields, we use a Lorentzian function to find the resonance peak of the signal. The resonance peaks then are used as input of eq. 1. With this equation we can fit the charge transition as a function of the magnetic field (black curve).

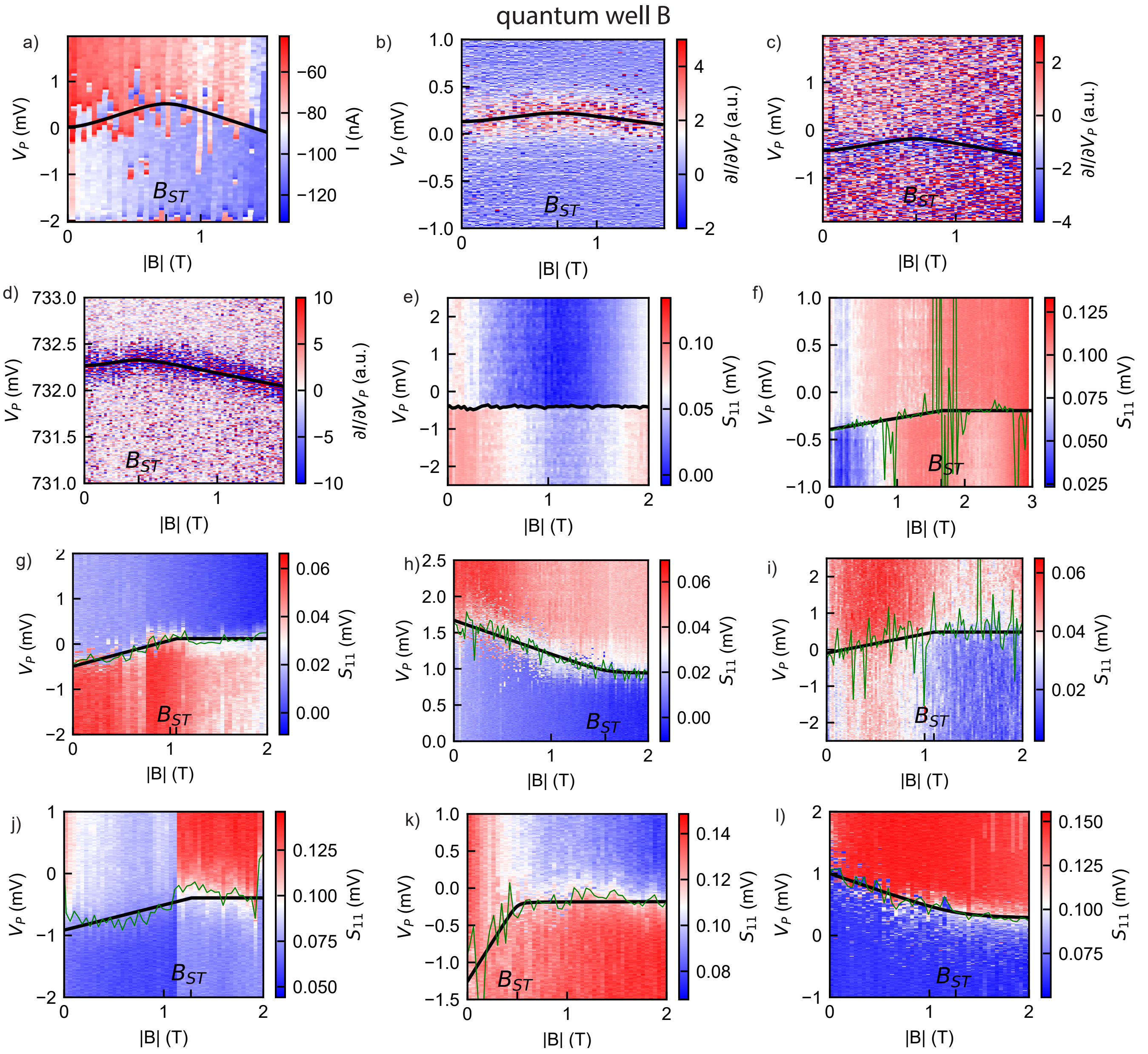

Supplementary Figure 6. Magnetospectroscopy of quantum dots fabricated on quantum well B. V_P is the gate voltage applied to the plunger gate forming the quantum dot. For clarity, we subtract from V_P in panels a) - l) an offset that depends is on the quantum dot being measured. a) -d) Magnetospectroscopy data measured along the $N = 1 \rightarrow 2$ transition of four different quantum dots on two different samples in quantum well B. The signal is measured by monitoring **a**, the current I through a nearby charge sensor, or **b** - **d** by monitoring the derivative of the current $\frac{\partial I}{\partial V}$ through a nearby charge sensor. **a** - **d**, To extract the inflection point of the electron charge transition, we fit the signal of the detuning for every magnetic field to eq. (2) from Ref. [9]. The inflection points then are used as input of eq. 1. With this equation we can fit the charge transition as a function of the magnetic field (black curve). e) - l) Magnetospectroscopy data measured along the $N = (1, 1) \rightarrow (2, 0)$ transition of eight different quantum dots on two different samples in quantum well B. The signal is measured by monitoring the reflected amplitude of the rf readout signal through a nearby charge sensor. To extract the inflection point of the electron charge transition, we fit the signal of the detuning for every magnetic field to eq. (2) from Ref. [9]. Here we superimpose the inflection points as green curves, to help the reader to follow the charge transitions. To extract B_{ST} we use the crossing point of two linear fits (black solid lines) along the T_- and S_0-state. On top of these samples there is a micromagnet lowering the magnetic field strength at the center of the sample by up to 0.2 T corresponding to 23 μeV which is taken as a lower bound for measurable E_{ST}.

Stack	Wafer ID	database processing ID	Figure	device ID	transition	B_{ST} (T)	E_{ST} (μeV)	d_p (nm)
QW A	QT428	DEMO 13	S4a	D1 2-dot, P2	(0,1) → (0,2)	1.11	129±1.1	50
QW A	QT428	DEMO 13	S4b	D1 2-dot, P1	(0,1) → (0,2)	0.42	49.4±2.2	50
QW A	QT428	DEMO 21	S4c	D2 2-dot, P1	(0,1) → (0,2)	0.83	96.6±6.3	50
QW A	QT428	DEMO 21	S4d	D2 2-dot, P2	(0,1) → (0,2)	1.47	170.4±9.0	50
QW A	QT428	DEMO 15	S4e	D3 2-dot, P1	(0,1) → (0,2)	1.52	176.3±13.4	50
QW A	QT428	DEMO 15	1f, S4f	D3 2-dot, P2	(0,1) → (0,2)	1.57	182.3±5.8	50
QW A	QT539	SQ19-193-1-3-03	S4, g	D4 2-dot, P1	(0,1) → (0,2)	0.31	35.7±5.9	50
QW A	QT539	SQ19-193-1-3-03	S4, h	D4 2-dot, P2	(0,1) → (0,2)	0.45	52.6±0.8	50
QW A	QT539	SQ19-193-1-3-04	S4, i	D5 2-dot, P1	(0,1) → (0,2)	0.9	104±1.6	50
QW A	QT539	SQ19-193-1-3-04	S4, j	D5 2-dot, P2	(0,1) → (0,2)	0.69	79.6±2.0	50
QW B	QT592	SQ20-20-5-25-2	S5, a	D1 5-dot, P4	(0,1) → (0,2)	0.74	85.7±2.0	40
QW B	QT592	SQ20-20-5-25-2	S5, b	D1 5-dot, P1	(0,1) → (0,2)	0.71	82.1±3.7	40
QW B	QT592	SQ20-20-5-25-2	S5, c	D1 5-dot, P2	(0,1) → (0,2)	0.7	81.7±10.1	40
QW B	QT553	SQ19-228-2-44-2	S5, d	D6 2-dot, P2	(0,1) → (0,2)	0.41	47.2±3.68	50
QW B	QT592	SQ20-20-5-18-4	S5, e	D1 6-dot, P3	(1,1) → (0,2)	0	0±0	50
QW B	QT592	SQ20-20-5-18-4	S5, f	D1 6-dot, P4	(1,1) → (0,2)	1.73	191.5±13.2	50
QW B	QT637	SQ20-205-2-12	S5, g	D2 6-dot, P1	(1,1) → (0,2)	1.06	123.1±8.9	40
QW B	QT637	SQ20-205-2-12	S5, h	D2 6-dot, P2	f (1,1) → (0,2)	1.56	180.5±9.7	40
QW B	QT637	SQ20-205-2-12	S5, i	D2 6-dot, P3	(1,1) → (0,2)	1.1	126.8±33.6	40
QW B	QT637	SQ20-205-2-12	S5, j	D2 6-dot, P4	(1,1) → (0,2)	1.27	147.3±15.7	40
QW B	QT637	SQ20-205-2-12	S5, k	D2 6-dot, P5	(1,1) → (0,2)	0.5	57.9±13.5	40
QW B	QT637	SQ20-205-2-12	S5, l	D2 6-dot, P6	(1,1) → (0,2)	1.25	144.6±19.1	40

Supplementary Table S1. Summary of quantum dot valley splitting measurements. Among all devices measured, in one case (data point $E_{\mathrm{ST}} = 0$ μeV) we did not observe in magnetospectroscopy the signature kink associated with valley splitting. This indicates a very small valley splitting, below the lower bound of about 23 μeV set by our experimental measurement conditions . While very small valley splitting values are within the predicted theoretical distributions in the main text, previous theories[10] suggest that they could also originate from the presence of an atomic step within the quantum dot.

2. MATERIAL CHARACTERIZATION

a. Atom Probe Tomography analysis of interfaces

Atom Probe analysis (APT) of the interfaces is done in 5 steps. All of them explained in detail below. First, the entire measurement is reconstructed using the standard reconstruction algorithms [11]. Second, a cube approximately representing the size of an electrical defined quantum in the x,y-plane and comfortably comprising the entire quantum well in the z-direction/depth-direction is extracted from the reconstructed data. This is done to have comparable sizes for each measurement, to limit the known reconstruction artefacts of APT [12] and to enable a direct comparison to simulations in step 5. Third, the three-dimensional point cloud created in the usual APT reconstruction [11] is tessellated using a Voronoi tessellation [13, 14]. The Voronoi tessellation is used for all subsequent steps. It can be viewed as a smoothing operation that "spreads out" the detected ions/atoms to a finite volume rather than representing them as zero-dimensional points. Forth, a x,y-grid is defined on the cube and for each cell of the grid a profile based on the Voronoi tessellation along the z-axis is created that is than fitted with a sigmoid function. The collection of sigmoid functions is then used to represent the interface and calculate the interface positions as well as the isoconcentration surfaces. Fifth, the profile extracted from the Voronoi grid of the entire cube is used to create a model structure with the known crystal structure of SiGe and a pseudo-random distribution of Si and Ge atoms in the x-y plane, enforcing the same profile along the depths direction as given by the Voronoi grid and the same percentage of atoms in the volume as expected from the detection efficiency of the Atom Probe (here: 80 % detection efficiency of the LEAP 5000XS). These model structures interface are then compared to the measurement results. All data treatment is done in Python 3.9 using numpy 1.20.3 and scipy 1.6.3.

b. Extraction of the cubes and Voronoi tessellation

The cubes are manually extracted from the reconstructed volume as exemplary shown in Fig. 7 a-b). After a cube containing the quantum well with the approximate size of an electrically defined quantum dot (∼ 30x30x20 nm) is extracted a Voronoi tessellation is performed on the point cloud representing APT data inside the cube. A result of such a tessellation in exemplary shown in Fig. 7 c).

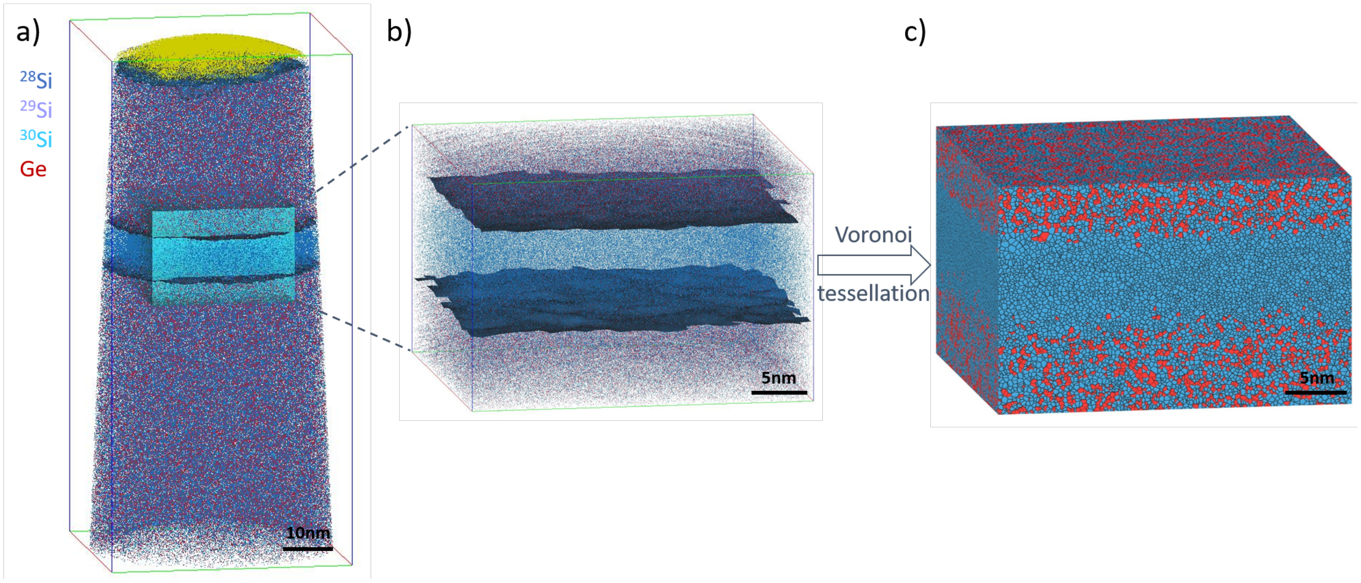

Supplementary Figure 7. Visualization of the extraction (a) of the cube (b) from the full data set (a) and Voronoi tessellation of the cube (c)

c. Construction of the interface

Interfaces are constructed based on the Voronoi tessellated data sets. The process is depicted in Fig. 8. A grid is created in the x,y-plane of the tessellated data set (Fig. 8 a-b). For each cell of the grid a one-dimensional profile along the z-axis is generated using the tessellation. As opposed to “regular” APT data [11] where profiles are created utilizing small bins along the z-axis and concentrations are then calculated from the ions/atoms within the bin [15] Chapter 7, the profiles on the tessellated data are created by a set of cutting planes. The process works by cutting the tessellation at each depth and use every ion/atom whose volume is cut as part of the plane and hence have it contribute to the concentration measured within that plane and at that depth.This can be viewed as a smoothing operation that spreads out the detected ions/atoms to a finite volume.

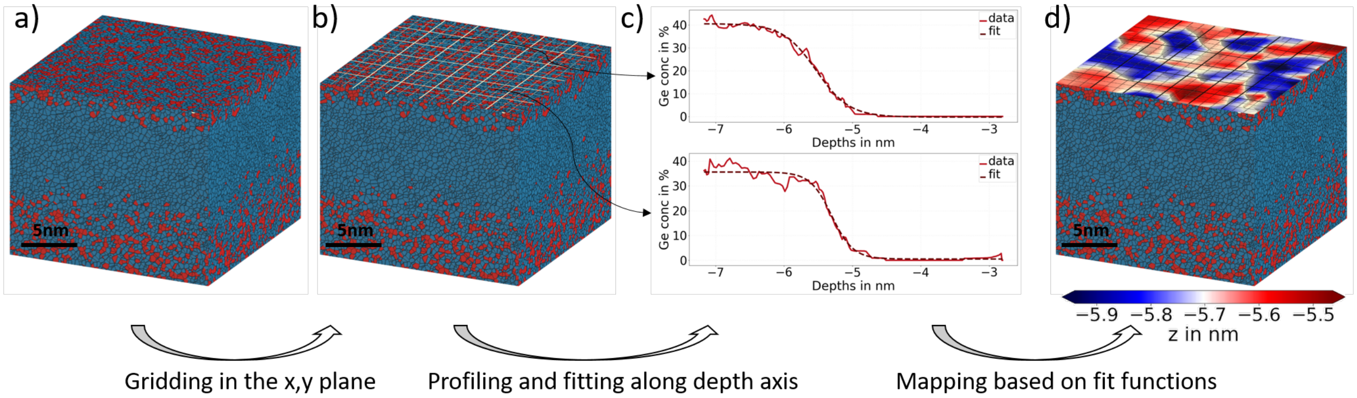

Supplementary Figure 8. Creation of a map from the Voronoi tessellated cube (a) by applying an x,y-grid (b) and fitting of profiles along z-axis with a sigmoid function in each cell (c). The profiles can then be used to calculate the position of e.g. the 25 % Germanium isoconcentration surface (d).

Each x,y-cell (typically 3x3 nm wide spaced 1 nm apart and hence partially overlapping) generates a profile and is then fitted using sigmoid function [16] as shown in Fig. 8 c). The sigmoid functions are then used to represent the interface in the following way:

- The inflection point of the sigmoid represents the position of the interface in each cell (Fig. 8 d)
- Isoconcentration surfaces [15] Chapter 6.3.2 are created by plotting the position where the sigmoid of each cell reaches the respective concentration

Fig. 9 and 10 show examples of the interface positions maps and isoconcentration surface maps generated in this way for the top and bottom interfaces of a QW A and a QW B sample. Note, that the data can now readily be used to calculate the average roughness and root mean square roughness in the usual way [17].

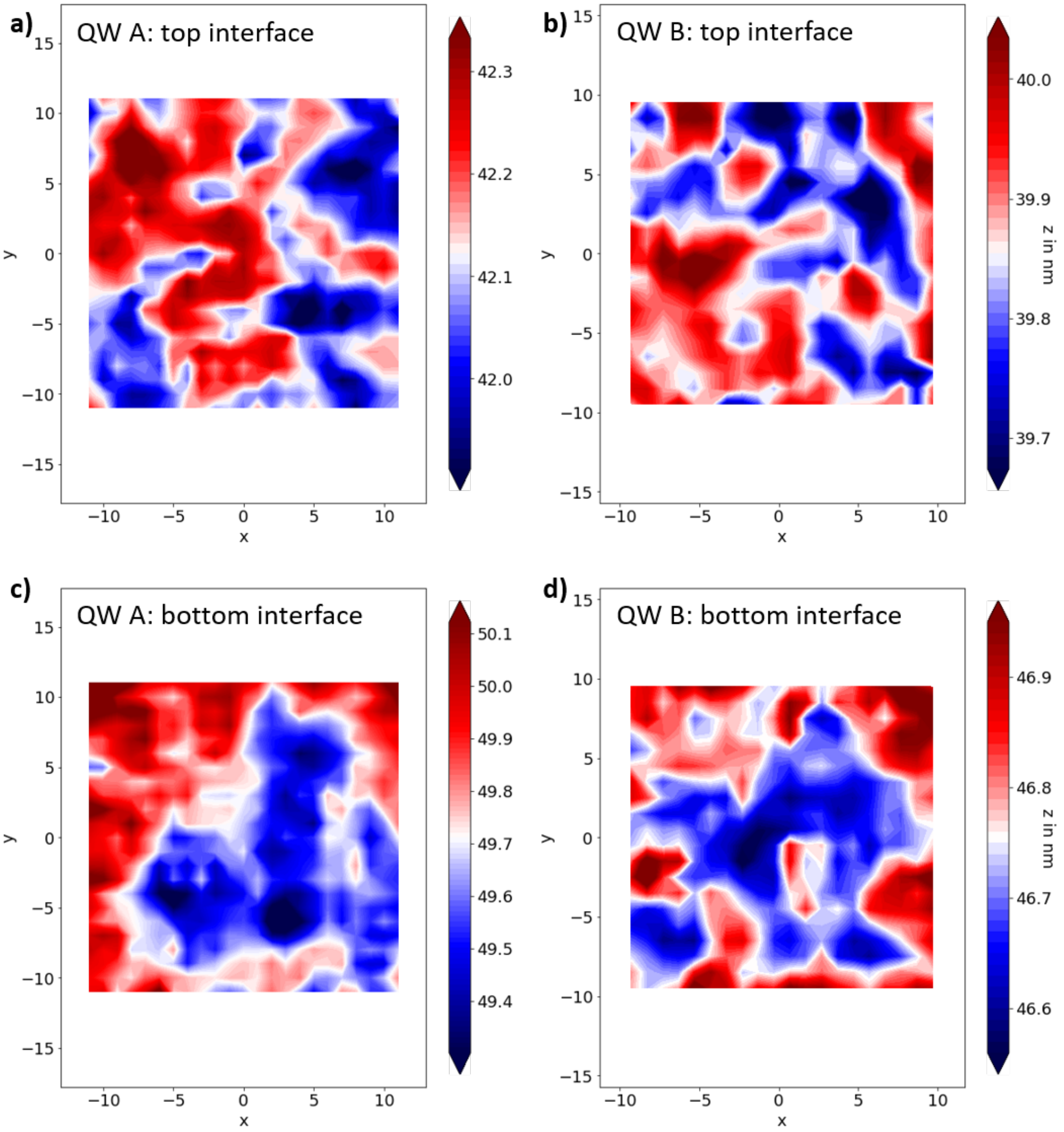

Supplementary Figure 9. Examples of position maps of top (a, b) and bottom (c, d) Germanium interfaces for both Quantum wells A and B. For each cell the depth plotted on the map is extracted from inflection point the sigmoid fit to the profile extracted from the cell (Fig. 8 b-c).

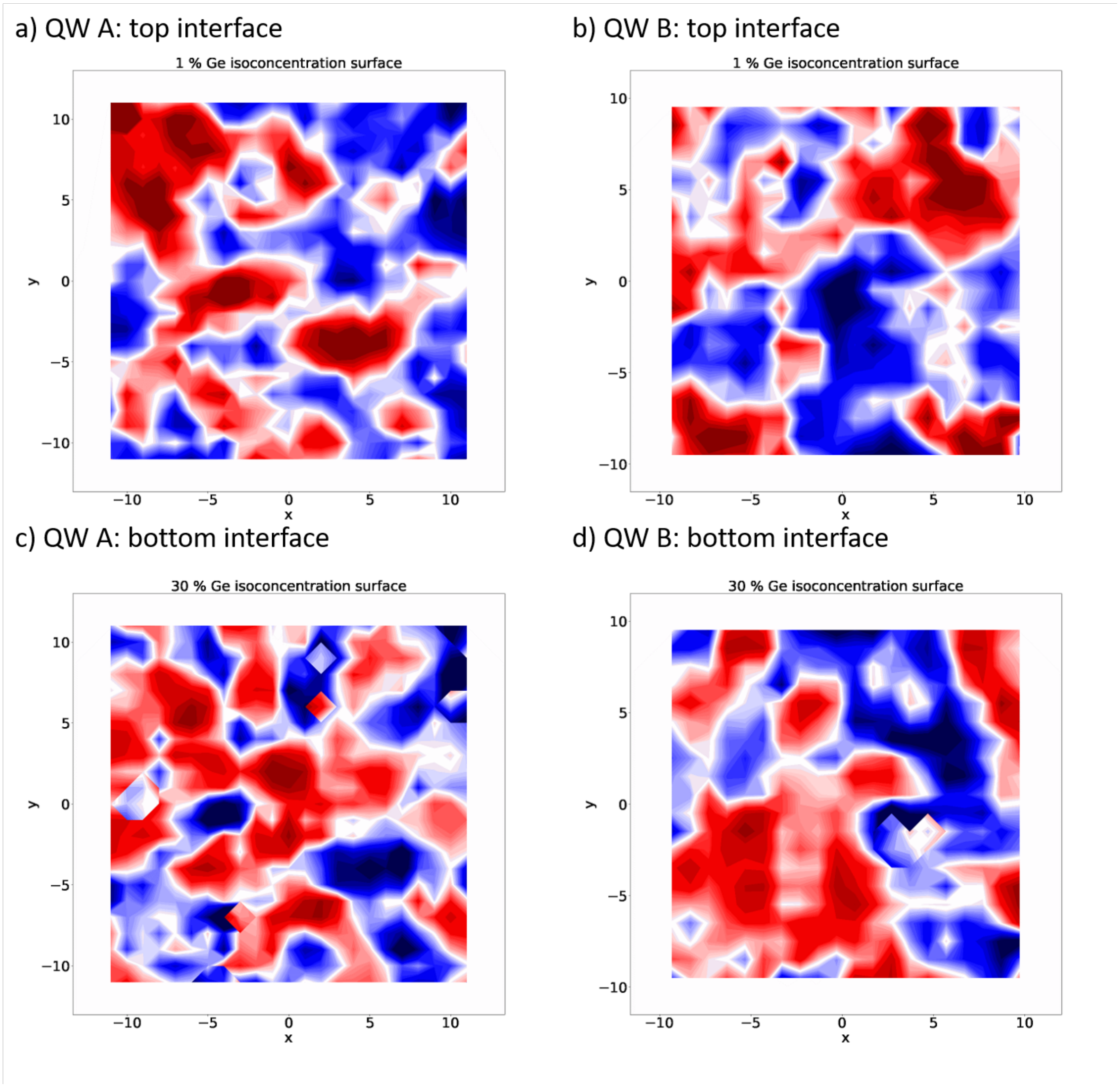

Supplementary Figure 10. Example of Germanium isoconcentration surfaces or the top (a, b) and bottom (c, d) interfaces of both Quantum Wells a and B. The plots reported here show one particular isosurface, 1% in a, b and 30% in c,d. As before the depth for each map can be extracted from the sigmoid fits to the profile in each cell.

d. Generating model data

Model data are generated based on the known crystal properties of $Si_{66.5}Ge_{33.5}$. A crystal of the same size as the cubes extracted from the data ($\sim$ 30x30x20 nm) is generated digitally and then 20 % of the atoms in the crystal are pseudo-randomly removed to account for the detection efficiency of the LEAP5000XS system used in the APT analysis.

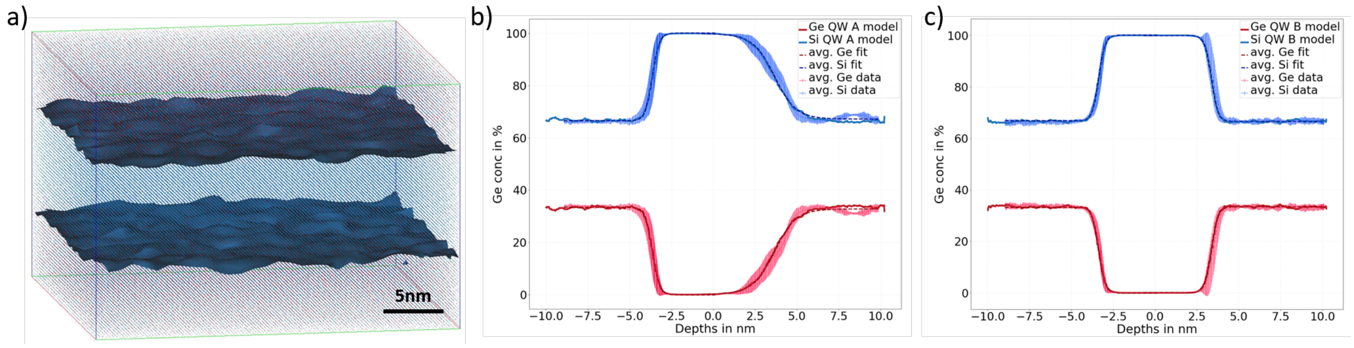

Supplementary Figure 11. Example of a crystalline cube of QW A (a) and a comparison of the average profiles of the measured quantum wells (see Fig. 2c of the main text) and profiles from a generated cube of Quantum Well A (b) and Quantum Well C (c).

Along the depth axis of the cube the average measured APT profile of the Si and Ge concentration of QW A and QW B as shown in Fig. 2c) of the main text is enforced. The result of the generation of such a cube for QW A and the comparison of the depth profile extracted from a cube of QWA and QW B to the average profile of QW A and QW B respectively are shown in Fig. 11. In Fig. 12 interface position maps of these model structures are shown. They should be compared to Fig. 9 where the same maps are extracted from measured data sets. The root mean square roughness as measured from the model is compared to the data measured from the APT data in Fig 2 of the main text.

Note: the animation in the file Supplementary_ Movie_1.m4v shows for the top interface of quantum well B (for increasing Ge concentration) the deviation of each isosurface tile position from the isosurface's average position. Here we benchmark the experimental data from our APT analysis (at each frame of the animation) against average and min-max range covered by 100 random models.

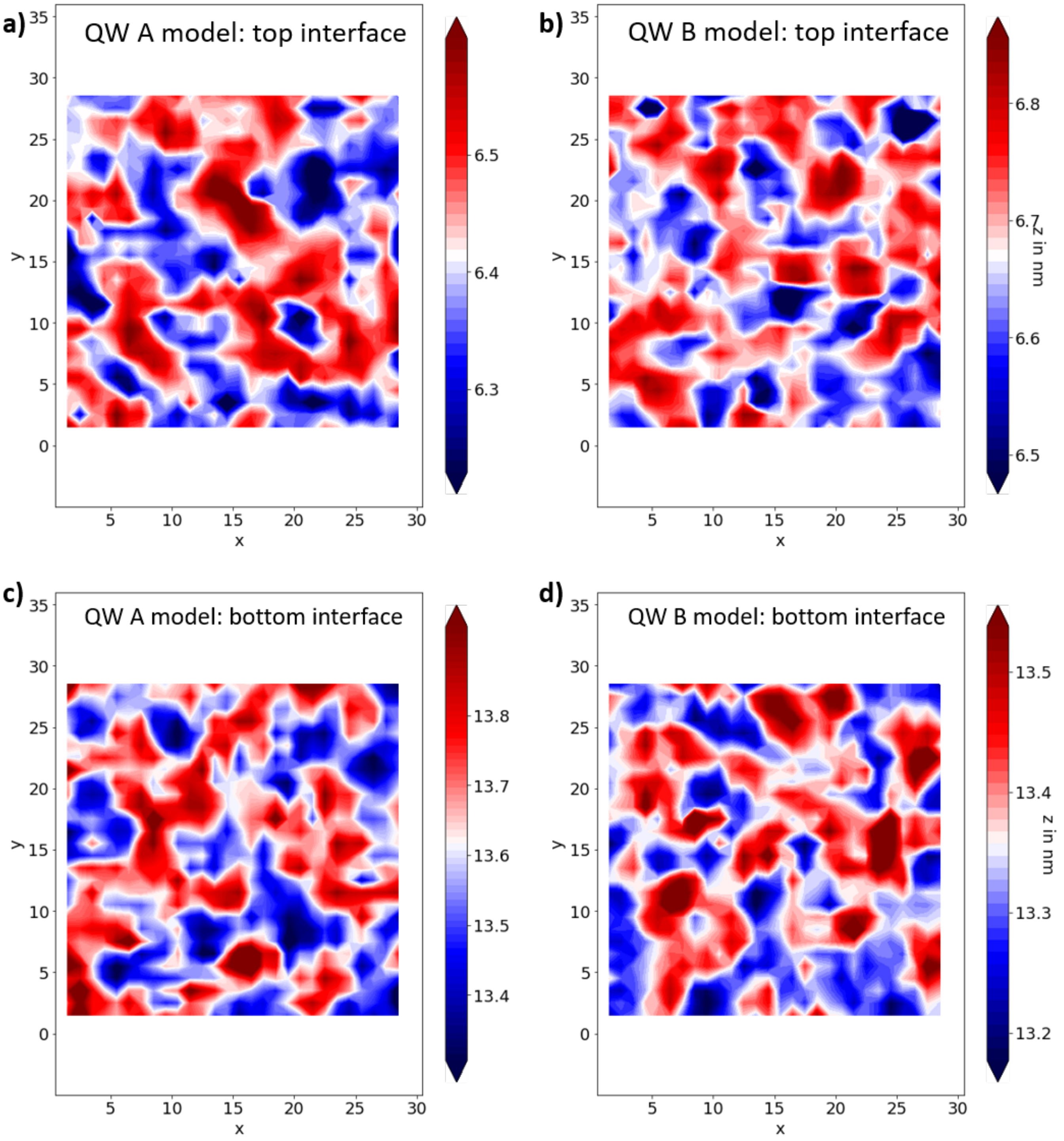

Supplementary Figure 12. Examples of position maps of top (a, b) and bottom (c, d) Germanium interfaces for model data sets of both Quantum wells A and B. As in Fig. 9 the depth plotted on the map is extracted from the inflection point of the sigmoid fit for the profile along the depth axis generated in each cell (Fig. 8 b-c).

e. Atomic steps, Quantum well width, and bottom interfaces

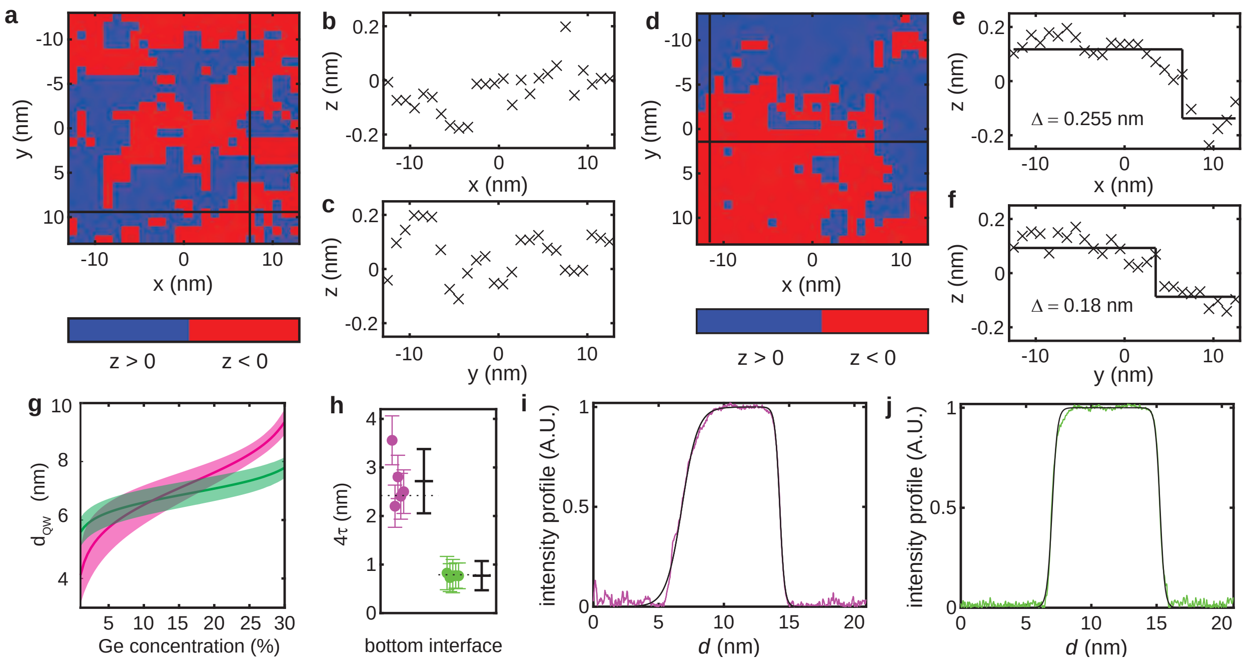

Supplementary Figure 13. **a** 10% isoconcentration surface from a Stack A sample without step. Blue areas are below and red areas above the average height (defined as $z = 0$) of the isoconcentration surface. The black lines are the positions of the line cuts in **b** and **c**. **b** line cut along the x direction of the isoconcentration surface. in **a**. **c** line cut along the y-direction of the isoconcentration surface. The z-position randomly oscillates around the mean value. **d** 10% isoconcentration surface from a Stack A sample with clear spatial division of the blue and red areas. The black lines are the positions of the line cuts in **e** and **f**. **e** line cut along the x-direction of **d**. A step with height $\Delta = 0.255$ nm occurs at $x = 7$ nm, corresponding to approximately 2 monoatomic layers. The black line represents the Heavyside step function with the highest C and the step height is determined by taking average z-position of the line cut before and after the step. **f** line cut along the y-direction of **c**. A step with $\Delta = 0.18$ nm occurs at $x = 3$ nm, corresponding to approximately 1.5 monoatomic layers. The black line represents the average z-position before and after the step. **g**, Average width of quantum well A (magenta line) and B (green line) as a function of the Ge concentration of the isoconcentration surfaces. Shaded areas represent the standard deviation of the quantum wells. **h**, Statistical analysis of the bottom 4τ interface widths derived from the fitting the data for quantum well A (magenta) and quantum well B (green). Black crosses are the mean and standard deviation for data from the different APT samples, highlighting the uniformity of the interfaces. **i**, **j**, HAADF-STEM intensity profile for stack A and B (magenta and green line, respectively) along the heterostructure growth direction (see TEMs in the main section). The black lines are fits of the data in the interface regions, using a sigmoid function.

To evaluate the presence of atomic steps from isoconcentration surfaces, we consider one-dimensional line cuts along the x- and y-axis of an isosurface. If a line cut crosses an atomic step along the isosurface, the line cut should resemble a Heavyside step function H:

$$H(x - x_s) = h_0 + \begin{cases} -a/2, & \text{for } x < x_s. \\ a/2, & \text{for } x \geq x_s. \end{cases} \tag{4}$$

where a is the step height, x_s is the step position and the offset h_0. To quantify the resemblance between a line cut and the step function, we determine the correlation coefficient C between the two with:

$$C = \frac{\sum_k (z_k - \bar{z})(h_k - \bar{h})}{\sqrt{\sum_k (z_k - \bar{z})^2}\sqrt{\sum_k (h_k - \bar{h})^2}} \tag{5}$$

where z_k are the z-values of the line cut, $\bar{z}$ is the mean value of the line cut, k is the index of the, h_k are the values of the step function, and $\bar{h}$ the mean value of the step function. If $C \geq 0.75$ we consider the linecut to represent a step. We subsequently can determine a by taking the difference between the two plateaux $\Delta = \bar{z}_{k+} - \bar{z}_{k-}$, where $\bar{z}_{k+}$ and $\bar{z}_{k-}$ are the average z-position before and after x_s, respectively.

f. SIMS and crosshatch pattern

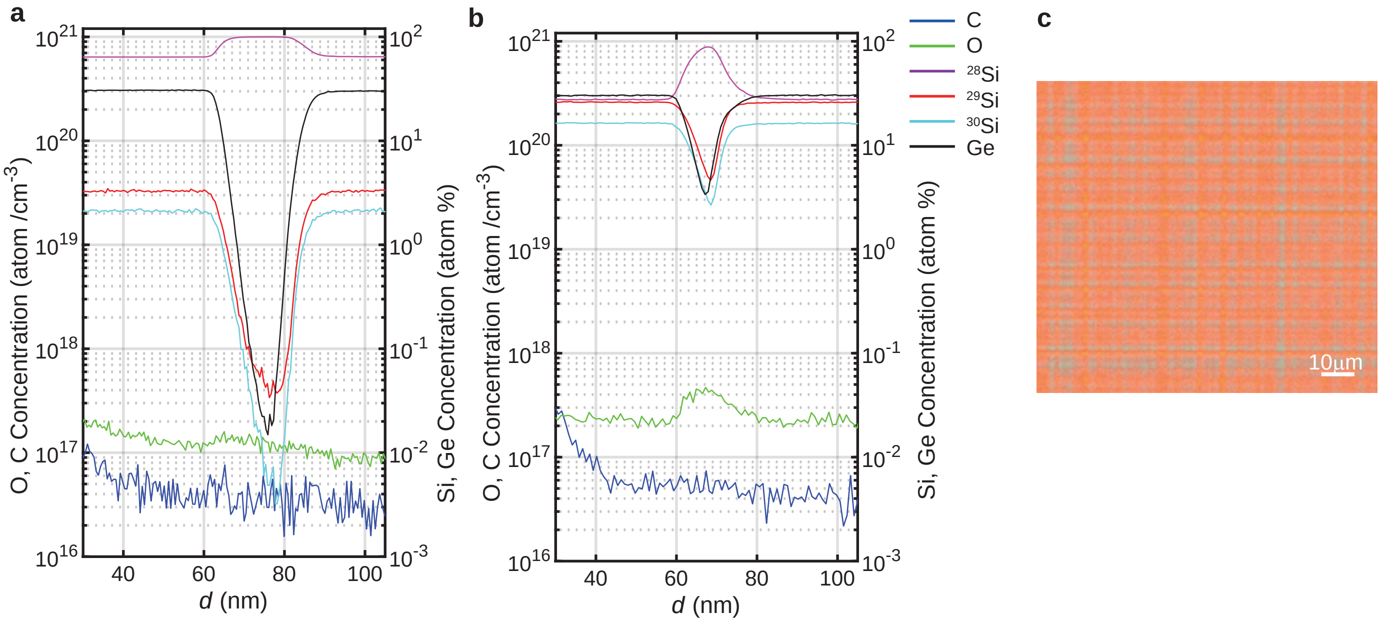

Supplementary Figure 14. **a**, **b**, Depth concentration SIMS profile of quantum well A and quantum well B respectively. Analyzed elements are ^{28}Si (red), ^{29}Si (blue), ^{30}Si (purple), Ge (black), oxygen (green) and carbon (blue). In quantum well A both carbon and oxygen concentrations are below their respective detection limits of 3×10^{16} cm^{-3} and 1×10^{17} cm^{-3}. In quantum well A only carbon is below the detection limits, while there is a residual oxygen content of 4×10^{17} cm^{-3} in the quantum well. **c** typical cross-hatch pattern from the surface of the wafers.

3. THEORETICAL MODEL

All numerical simulations and analysis in this section were performed in Python, using the open-source libraries NumPy, SciPy, and Matplotlib.

a. Tight-binding model

In our analysis, we use the one-dimensional two-band tight-binding model of Boykin *et al.* [18]. Here, the nearest-neighbor and next-nearest-neighbor hopping amplitudes, chosen to yield the correct valley minimum wavevector and out-of-plane effective mass, are $v = 0.683$ and $u = 0.612$, respectively. The onsite energy ε is the sum of the contributions from the electrostatic potential energy eEz, for a vertical electric field $E = 0.0125$ V/nm, and the quantum well confinement potential $U(z)$. The conduction band offset is taken to be a linear interpolation:

$$\Delta E_c = (x_w - x_s)\left[\frac{x_w}{1-x_s}\Delta E^{\mathrm{Si}}_{\Delta_2}(x_s) - \frac{1-x_w}{x_s}\Delta E^{\mathrm{Ge}}_{\Delta_2}(x_s)\right], \tag{6}$$

where x_w is the Si concentration inside the well and x_s is the Si concentration in the quantum well barriers (the substrate). The functions $\Delta E^{\mathrm{Si(Ge)}}_{\Delta_2}(x)$ describe the Δ_2 conduction band offsets for strained Si (Ge) grown on an unstrained $\mathrm{Si}_x\mathrm{Ge}_{1-x}$ substrate. These functions are approximately linear in x over their entire range, with limiting behaviors [19]

$$\begin{aligned}\Delta E^{\mathrm{Si}}_{\Delta_2}(x) &\approx -0.502(1-x)\ (\mathrm{eV}),\\ \Delta E^{\mathrm{Ge}}_{\Delta_2}(x) &\approx 0.743 - 0.625(1-x)\ (\mathrm{eV}),\end{aligned} \tag{7}$$

when $x \to 0$. The linearization scheme employed here agrees well with theoretical calculations [19].

As described in Methods, for our one-dimensional model, we determine the quantum well potential $U(z_l)$ at each atomic layer position z_l by linearly interpolating the conduction band offset between the barriers and the bottom of the well, yielding

$$U(z_l) = \frac{x_l^d - x_s}{x_w - x_s}\Delta E_c, \tag{8}$$

where x_l^d is the Si concentration at layer l, averaged over the lateral probability distribution of the quantum dot wavefunction, as explained in subsection c, below. To simulate many different quantum wells, we allow for random fluctuations of x_l^d, due to the finite size of a quantum dot. Below, we derive the statistical distribution of x_l^d, as reported in Eq. (18).

b. Comparison with NEMO-3D

The two-band tight-binding model has the advantage of being computationally inexpensive, allowing us to perform many random samplings, to obtain accurate statistics. To validate the model, we compare our results to those of a more sophisticated 20-band $\mathrm{sp^3d^5s^*}$ NEMO-3D tight-binding model (including spin) [20]. NEMO-3D heterostructures are generated atom by atom. Each atom is randomly chosen to be either Si or Ge, with the probability of choosing Si given by the concentration profile $\bar{x}_l$ for a given atomic layer l. The valley splitting is computed as the energy difference between the two lowest conduction states.

First, we consider the quantum well confinement profile shown in the inset of Fig. 15(c) for a quantum dot with a lateral parabolic confinement potential, corresponding to an orbital splitting of $\hbar\omega = 2.83$ meV, and a vertical electric field of $E = 0.0125$ V/nm. Note that in the two-band model, the resulting three-dimensional wavefunction is simply used to obtain a set of weighted one-dimensional layer concentrations, x_l^d. The results of these NEMO-3D simulations are plotted as histograms in Fig. 15(a), while the corresponding results from the two-band model are plotted in Fig. 15(b). Although fewer random samples are obtained in the NEMO-3D case, due to computational constraints, the two distributions appear to agree well. The NEMO-3D distribution is found to have a mean of 87.4 μeV and a standard deviation of 50.1 μeV, while the two-band distribution is found to have a mean of 104.7 μeV and a standard deviation of 55.7 μeV. Qualitatively, the two distributions have similar shapes. NEMO valley splittings are on average

slightly smaller, which is consistent with previous results [18].

To analyze the relationship between 2-band and NEMO simulations in more detail, we compute E_v with the 1D 2-band model using the same 60 heterostructures we used in Fig. 15(a). To do so, we took the heterostructure, computed the weighted average Si concentration at each layer, and plugged it into the 1D 2-band model. We assumed the wavefunction was in the ground state of a 2D isotropic harmonic oscillator potential, with characteristic energy $\hbar\omega = 2.83$ meV. Resulting valley splittings from NEMO (E_v^{NEMO}) and the 2-band model (E_v^{TB}) are plotted against each other in Fig. 15(c). We can see that there is a clear, strong linear correlation between the two. Fitting these data to the relationship $E_v^{\mathrm{NEMO}} = kE_v^{\mathrm{TB}}$, we find $k = 0.86$ with standard error 0.018. Again, the fact that NEMO valley splittings are slightly less than 2-band TB valley splittings is consistent with prior results [18].

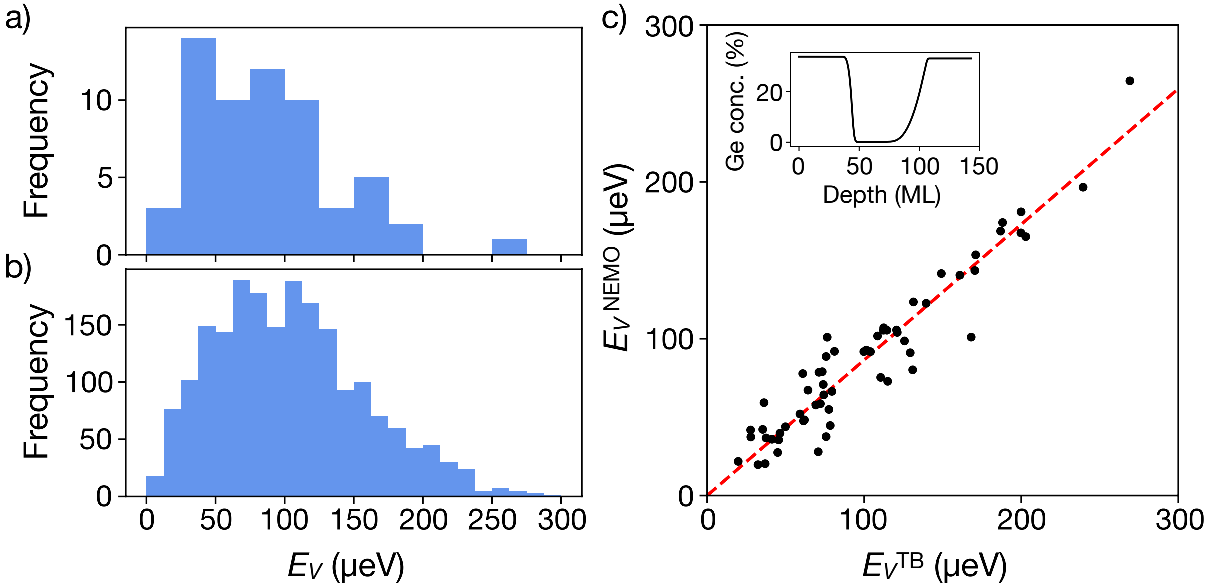

Supplementary Figure 15. A comparison of simulations with NEMO-3D and the 1-dimensional 2-band tight-binding model. (a) Histogram of 60 randomized valley-splitting simulations using NEMO-3D. (b) Histogram of 2,000 randomized valley-splitting simulations using the one-dimensional two-band tight-binding model. (c) We directly compare simulations using NEMO-3D and the 2-band model for the 60 heterostructures shown in (a). For each heterostructure, we plot its valley splitting with the 2-band model (E_v^{TB}) against it's valley splitting with NEMO (E_v^{NEMO}). The red dashed line shows the fit $E_v^{\mathrm{NEMO}} = kE_v^{\mathrm{TB}}$ with $k = 0.86$. All simulations assume an orbital splitting of $\hbar\omega = 2.83$ meV, and a vertical electric field of $E = 0.0125$ V/nm.

Next, we analyze the relationship between 2-band and NEMO simulations as a function of the Ge content in the well. Using the same heterostructures for both 2-band and NEMO simulations, we compute valley splittings with both models, performing 20 simulations for quantum wells with each of 0%, 5%, and 10% Ge. The resulting data are shown in Fig. 16(a). Again, there is a strong linear correlation between E_v^{TB} and E_v^{NEMO}. This correlation is tightest for 0% Ge, but still clear with Ge in the well. Again, we fit all the data to $E_v^{\mathrm{NEMO}} = kE_v^{\mathrm{TB}}$, finding $k = 0.76$ with standard error 0.053, indicated in the inset of Fig. 16(a). We also perform the same fit for each of the 0%, 5%, and 10% Ge data individually, also shown in the inset of Fig. 16(a), and we find that the resulting k values are not significantly different. We also note that these fit parameters are different than those from Fig. 15(c), indicating that there may be some interface-dependence for k. For these simulations, we used dots with characteristic orbital energy $\hbar\omega = 2$ meV and vertical field $E = 0.0125$ V/nm. We use 80 ML wide wells and sigmoid interfaces with widths $4\tau = 10$ ML.

Fig. 16(b) shows the mean and 25-75 percentile range of the valley splittings computed with NEMO as a function of the quantum well Ge concentration. As presented in the main text, the average valley splitting clearly grows with increasing Ge content. This is a nice validation of the main results obtained with the simple 1D model.

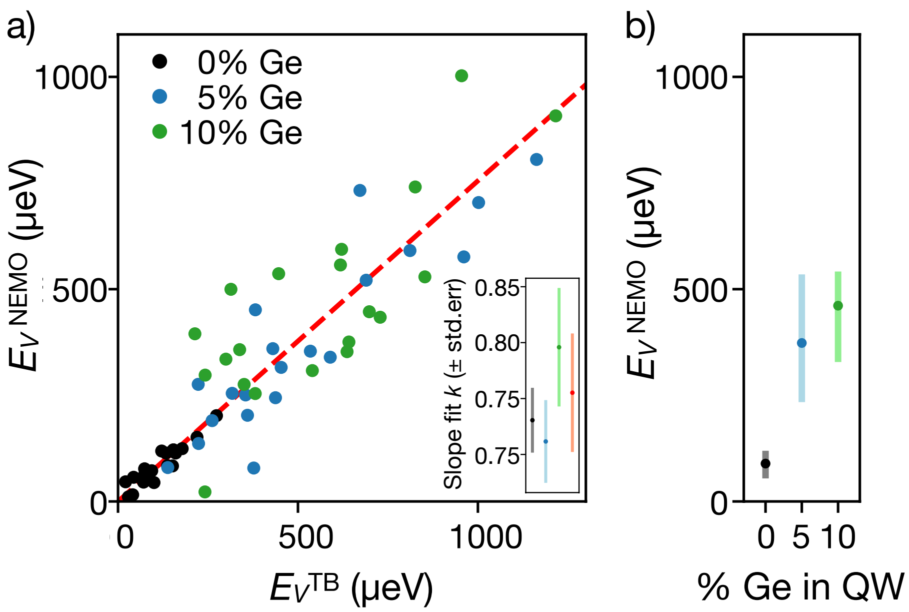

Supplementary Figure 16. (a) A comparison of valley splittings computed with the 2-band TB model (E_v^{TB}) and NEMO-3D (E_v^{NEMO}). Valley splittings for each point were computed using the same heterostructure on both methods. Black, blue, and green data correspond to quantum wells with 0%, 5%, and 10% Ge, respectively. The red dashed line indicates the best fit $E_v^{\mathrm{NEMO}} = kE_v^{\mathrm{TB}}$, where $k = 0.76$ with standard error 0.026, shown in red in the inset. Also shown in the inset are fit values k and their standard errors for each subset of data individually. (b) The mean and 25-75 percentile range of E_v^{NEMO} for quantum wells with 0%, 5%, and 10% Ge. For all simulations, we use quantum wells with widths of 80 ML and interface widths of $4\tau = 10$ ML. We assume a parabolic confinement potential with strength $\hbar\omega = 2$ meV and vertical field $E = 0.0125$ V/nm.

c. Statistical distribution of intervalley couplings

Here, we examine the derivation of the statistical distribution of the intervalley coupling matrix element in more detail. From Methods, we have the intervalley coupling matrix element

$$\Delta = \frac{a_0}{4} \sum_l e^{-2ik_0 z_l} \frac{x_l^d - x_s}{x_w - x_s} \Delta E_c |\psi_{\mathrm{env}}(z_l)|^2, \tag{9}$$

where the valley splitting $E_v = 2|\Delta|$. As above, x_l^d represents the Si concentration in atomic layer l, weighted by the probability distribution of the electron charge density in the dot. We can split Eq. (9) into its deterministic and random contributions, using the definition $x_l^d = \bar{x}_l + \delta_l$, where $\bar{x}_l$ is the ideal, smooth concentration profile of the heterostructure and δ_l are the random fluctuations about this profile. The random contribution to the matrix element can then be expressed as

$$\delta\Delta = \frac{a_0 \Delta E_c}{4(x_w - x_s)} \sum_l e^{-2ik_0 z_l} \delta_l |\psi_{\mathrm{env}}(z_l)|^2. \tag{10}$$

We now calculate the variance of the matrix element. First, we note that the random fluctuations only occur in $\delta\Delta$, so that $\mathrm{Var}[\Delta] = \mathrm{Var}[\delta\Delta]$. Second, we recall that the variance of a complex random variable is the sum of the variances of its real and imaginary components. Third, since we assume the fluctuations in different layers are independent, the variance of the sum in Eq. (10) must be equal to the sum of the variances in each layer. In this way, we obtain

$$\mathrm{Var}[\Delta] = \left[\frac{a_0 \Delta E_c}{4(x_w - x_s)}\right]^2 \sum_l |\psi_{\mathrm{env}}(z_l)|^4 \mathrm{Var}[\delta_l]. \tag{11}$$

To compute $\mathrm{Var}[\delta_l]$, we note again that x_l^d represents the fluctuating Si concentration in layer l, weighted by the electron charge density. We first describe the weighting function, which is defined in a single two-dimensional layer, and is proportional to the squared amplitude of the wavefunction. In the lowest-subband approximation [21], the dot

wavefunction is separable, so the in-plane component of the wavefunction $\psi_t(x,y)$ does not depend on the layer index. For a circular, parabolically confined dot with orbital excitation energy $\hbar\omega_{\rm dot}$, the normalized wavefunction is given by $\psi_t(x,y) = (\pi a_{\rm dot}^2)^{-1/2}\exp(-r^2/2a_{\rm dot}^2)$, where the dot is taken to be centered at the origin, $a_{\rm dot} = \sqrt{\hbar/m_t\omega_{\rm dot}}$ is a characteristic dot dimension, $m_t = 0.19\,m_e$ is the transverse effective mass, and $r = \sqrt{x^2+y^2}$. Let us consider the dot weighting function $w(a)$, which is defined only at the atom locations $a \in A_l$, where A_l is the set of all atom positions in layer l. Since $w(a)$ is defined discretely, while $\psi_t(x,y)$ is continuous, their normalizations are different. To determine the normalization of $w(a)$, we require that $\sum_{a\in A_l} w(a) = 1$, which is analogous to the wavefunction normalization $\int_{-\infty}^{\infty} dx\,dy |\psi_t(x,y)|^2 = 1$. The correspondence between between the sum, $\sum_{a\in A_l}$, and the integral, $\int_{-\infty}^{\infty} dx\,dy$, must take into account the fact that there are two atoms in every two-dimensional unit cell of size $a_0 \times a_0$, for a diamond cubic crystal like Si. Normalizing over a single unit cell, the correct correspondence is therefore given by

$$\sum_{a\in A_l} \to \frac{2}{a_0^2}\int_{-\infty}^{\infty} dx\,dy. \tag{12}$$

Next, we define $w(a) = c|\psi_t(r_a)|^2$, where c is a proportionality constant, to be determined below, and $\mathbf{r}_a = (x_a, y_a)$ is the 2D coordinate location of atom a. Using the correspondence in Eq. (12), we obtain the appropriate normalization for $w(a)$:

$$w(a) = \frac{a_0^2}{2\pi a_{\rm dot}^2} e^{-r_a^2/a_{\rm dot}^2}. \tag{13}$$

Now, according to its definition, the weighted Si concentration in layer l is given by

$$x_l^d = \sum_{a\in A_l} \mathbb{1}\,[a = \text{Si}]\, w(a), \tag{14}$$

where the indicator function, $\mathbb{1}\,[a = \text{Si}]$, takes the value 1 if a is a Si atom and 0 otherwise. The fluctuating part of the concentration is given by $\delta_l = x_l^d - \bar{x}_l$. Calculating $\text{Var}\,[\delta_l]$, we then obtain

$$\begin{aligned}
\text{Var}\,[\delta_l] &= \text{Var}\left[\sum_{a\in A_l} \mathbb{1}\,[a = \text{Si}]\, w(a)\right] \\
&= \sum_{a\in A_l} w^2(a)\text{Var}\,[\mathbb{1}\,[a = \text{Si}]] \\
&= \bar{x}_l(1-\bar{x}_l)\sum_{a\in A_l} w^2(a).
\end{aligned} \tag{15}$$

Here, in the second line, we use the fact that the atoms in the random alloy are chosen independently. In the third line, we use the fact that each indicator function in the sum is a Bernoulli trial with variance given by $p(1-p)$ [22], where the probability of success is given by $p = \bar{x}_l$. Making use of Eqs. (12) and (13), we arrive at

$$\text{Var}[\delta_l] = \bar{x}_l(1-\bar{x}_l)\frac{a_0^2}{4\pi a_{\rm dot}^2}, \tag{16}$$

and

$$\text{Var}\,[\Delta] = \frac{1}{\pi}\left[\frac{a_0^2 \Delta E_c}{8a_{\rm dot}(x_w - x_s)}\right]^2 \sum_l |\psi_{\rm env}(z_l)|^4 \bar{x}_l(1-\bar{x}_l). \tag{17}$$

To complete the calculation of Var[Δ], we need to evaluate $\psi_{\rm env}(z_l)$. In this work, we compute $\psi_{\rm env}(z_l)$ numerically, for the ideal concentration profile $\bar{x}_l$, and the corresponding confinement potential $U(z_l)$ obtained from Eq. (8), by discretizing the Schrodinger equation on the atomic lattice sites z_l.

Although analytical methods can be used to characterize $x_l^d = \bar{x}_l + \delta_l$, as in Eq. (16), we also perform other types of randomized numerical simulations, as described in the main text. To do this, we could assign atoms in a 3D array, with each atom in layer l having the probability $\bar{x}_l$ of being silicon. The statistical properties of such an array derive from a binomial distribution. In principle, for real wave functions, such calculations could involve a very large number

of atoms, to accurately describe the wavefunction tails. Alternatively, we may consider a much smaller number atoms N_{eff}, for which

$$x_l^d \sim \frac{1}{N_{\text{eff}}} \text{Binom}\left(N_{\text{eff}}, \bar{x}_l\right), \tag{18}$$

where Binom (n, p) is the binomial distribution with n trials and probability of success p. The question now becomes, how should we determine N_{eff}? The answer is that N_{eff} should be chosen to yield the correct statistical properties for x_l^d, including its mean and variance. Using the known variance of the binomial distribution, $N_{\text{eff}}\,\bar{x}_l(1-\bar{x}_l)$, and comparing to Eq. (16), we see that we should choose $N_{\text{eff}} = 4\pi a_{\text{dot}}^2/a_0^2$. This corresponds to an effective dot radius of $r_{\text{eff}} = \sqrt{2}\,a_{\text{dot}} = \sqrt{2\hbar/m_t\omega_{\text{dot}}}$.

d. Statistical distribution of valley splittings

Since the intervalley coupling Δ is a complex random variable and is the sum of many independent random variables (corresponding to the different layers), it follows a 2D Gaussian distribution in the complex plane. In Eq. (17), we derived the variance of the intervalley coupling Δ due to concentration fluctuations. We now examine the distribution of valley splittings $E_v = 2|\Delta|$. The magnitudes of points sampled from a circular Gaussian distribution in the complex plane follow a Rice distribution [23], whose probability density function is given by

$$f_{\text{Rice}}(z|\nu,\sigma) = \frac{z}{\sigma^2} \exp\left(-\frac{z^2+\nu^2}{2\sigma^2}\right) I_0\left(\frac{z\nu}{\sigma^2}\right) \tag{19}$$

where ν is the distance from the origin to the center of the circular Gaussian, σ is the width of the Gaussian along one of its axes, and $I_0(y)$ is a modified Bessel function of the first kind. Since the Gaussian distribution for Δ is centered on the deterministic value Δ_0, the probability density function for valley splittings $f_{\text{Rice}}(E_z|\nu,\sigma)$ is centered at $\nu = 2|\Delta_0|$. The variances of the real and imaginary components are both given by $(1/2)\text{Var}\,[\delta\Delta]$, such that $\sigma = \sqrt{2}\sqrt{\text{Var}\,[\delta\Delta]}$.

e. Effects of interface width and QW Ge concentration on average valley splitting

Both the average Ge concentration and the width of the interface have an effect on the valley splitting in a quantum well. Here, we analyze the contributions of both. Figure 17(a) provides an extended version of Fig. 3(g) in the main text, showing the valley splitting distributions at different quantum well Ge concentrations and interface widths. The valley splittings at interface width $4\tau = 0$ are consistently large, regardless of quantum well Ge concentration, due to the large deterministic component for this (somewhat unphysical) geometry. For more realistic, nonzero interface widths, increasing the Ge concentration in the quantum well increases the valley splitting. At low Ge concentration, wider interfaces can actually increase average valley splitting, because the wavefunction sees layers with more Ge. Figure 17(b) provides an extended dataset, showing the average valley splitting at several combinations of interface width vs. Ge concentration in the well. For very narrow interfaces, E_v is large, regardless of the Ge concentration in the well. For wider interfaces, adding Ge to the well consistently boosts the valley splitting. The grey line in Fig. 17(b) delineates quantum wells for which $\geq 95\%$ of simulations have $E_v \geq 100\ \mu$eV (the large, upper-left portion of the plot). For realistic electric fields, we find that any well with $> 5\%$ Ge, regardless of interface width, should have $E_v \geq 100\ \mu$eV at least 95% of the time.

Figure 17(c) shows a sample wavefunction in a quantum well with an interface width of $4\tau = 20$ ML. The wavefunction is colored according to the Ge concentration in each layer, illustrating how a wide interface can expose the wavefunction to more layers with nonzero Ge content.

f. Effect of vertical electric field on average valley splitting

In this section, we investigate the effect of the vertical electric field on the valley splitting distributions in quantum well A and B. Fig. 18 shows the mean and 25-75 percentile range of 1000 1D 2-band tight-binding simulations of E_v with various vertical fields E_z, for both quantum wells A and B. Increasing the vertical field leads to larger mean E_v and larger spreads in E_v as the quantum dot wavefunction penetrates the top interface, thereby increasing the alloy disorder. That said, even wells with zero vertical field still show a sizeable spread in E_v due to alloy disorder. In this paper, we use $E_z = 0.0075$ V/nm because the resulting E_v distributions agree well with the data.

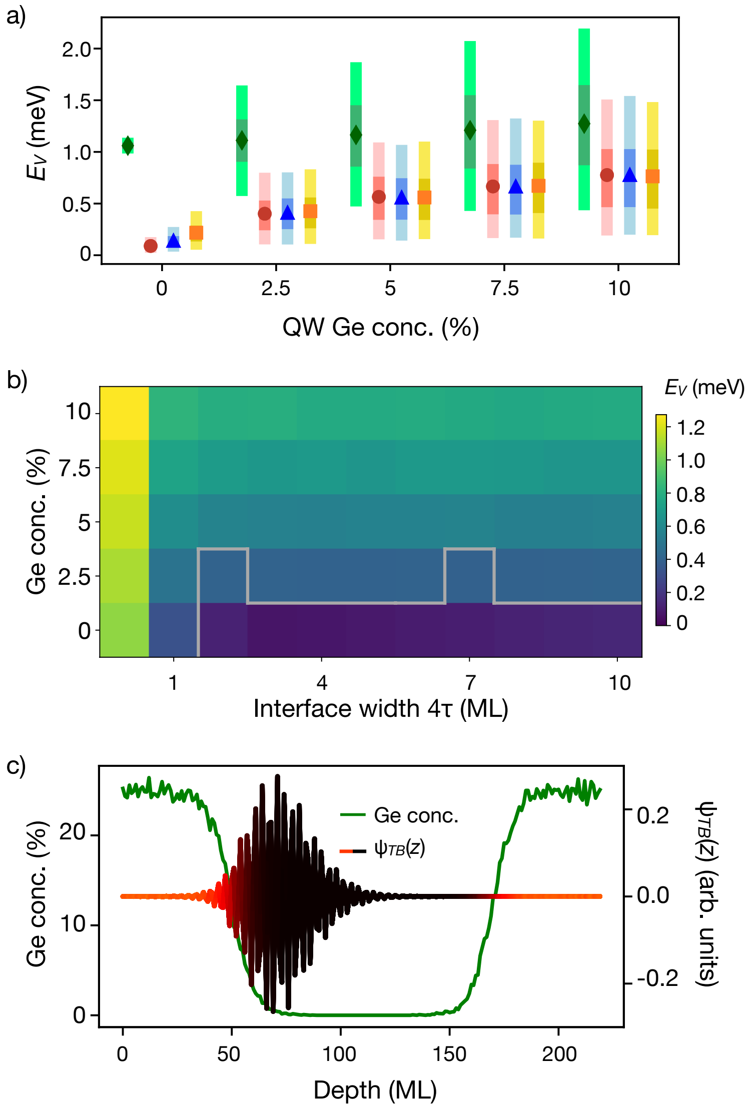

Supplementary Figure 17. Valley splitting simulations. (a) An extended version of Fig. 3(g) in the main text. Valley splitting distributions are shown for quantum well Ge concentrations from 0 to 10% and interface widths of $4\tau = 0$ ML (green diamonds), $4\tau = 5$ ML (red circles), $4\tau = 10$ ML (blue triangles), and $4\tau = 20$ ML (orange squares). The symbol represents the mean valley splitting, while the dark bars represent the 25-75 percentile range, and the light color bars represent the 5-95 percentile range. Each bar represents 2,000 simulations using the one-dimensional two-band tight-binding model. (b) The mean valley splitting is shown for a range of interface widths 4τ and quantum well Ge concentrations. Each pixel corresponds to 2,000 simulations using the one-dimensional two-band tight-binding model. All pixels above the grey line have $E_v \geq 100$ μeV in more than 95% of the simulations; all pixels below have $E_v \geq 100$ μeV less than 95% of the time. (c) A sample simulation of a quantum well with 0% Ge and an interface width of $4\tau = 20$ ML. The green curve shows the Ge concentration profile, and the red-black curve shows the ground state tight-binding wavefunction. The color of the wavefunction illustrates the Ge concentration of each layer, with red corresponding to high concentrations. Clearly, a significant portion of the wavefunction is found in a region with nonzero Ge content. All simulations in this figure were performed with an electric field of 0.0075 V/nm, for the quantum well depicted in Fig. 3(g) of the main text.

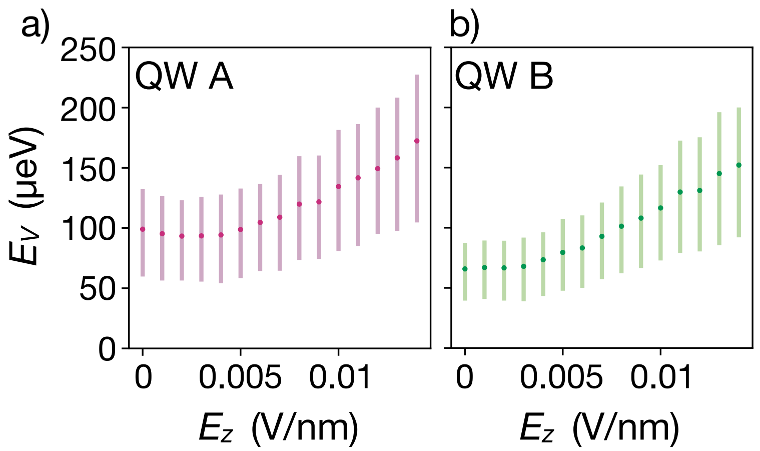

Supplementary Figure 18. The variation of the valley splitting distributions in quantum wells A (a) and B (b) as a function of the vertical electric field E_z. Each bar shows the mean and 25-75 percentile range of 1000 simulations using the 1D 2-band tight-binding model. We assume an orbital energy $\hbar\omega = 4.18$ meV.

[1] L. A. Tracy, E. H. Hwang, K. Eng, G. A. Ten Eyck, E. P. Nordberg, K. Childs, M. S. Carroll, M. P. Lilly, and S. Das Sarma, Physical Review B **79**, 235307 (2009).

[2] B. P. Wuetz, M. P. Losert, A. Tosato, M. Lodari, P. L. Bavdaz, L. Stehouwer, P. Amin, J. S. Clarke, S. N. Coppersmith, A. Sammak, *et al.*, Physical Review Letters **125**, 186801 (2020).

[3] E. J. Connors, J. Nelson, H. Qiao, L. F. Edge, and J. M. Nichol, Physical Review B **100**, 165305 (2019).

[4] A. Hollmann, T. Struck, V. Langrock, A. Schmidbauer, F. Schauer, T. Leonhardt, K. Sawano, H. Riemann, N. V. Abrosimov, D. Bougeard, *et al.*, Physical Review Applied **13**, 034068 (2020).

[5] D. Zajac, T. Hazard, X. Mi, K. Wang, and J. R. Petta, Applied Physics Letters **106**, 223507 (2015).

[6] X. Mi, C. G. Péterfalvi, G. Burkard, and J. R. Petta, Physical review letters **119**, 176803 (2017).

[7] J. Dodson, H. E. Ercan, J. Corrigan, M. Losert, N. Holman, T. McJunkin, L. Edge, M. Friesen, S. Coppersmith, and M. Eriksson, arXiv preprint arXiv:2103.14702 (2021).

[8] N. Samkharadze, G. Zheng, N. Kalhor, D. Brousse, A. Sammak, U. Mendes, A. Blais, G. Scappucci, and L. Vandersypen, Science **359**, 1123 (2018).

[9] L. DiCarlo, H. J. Lynch, A. C. Johnson, L. I. Childress, K. Crockett, C. M. Marcus, M. P. Hanson, and A. C. Gossard, Phys. Rev. Lett. **92**, 226801 (2004).

[10] M. Friesen, S. Chutia, C. Tahan, and S. N. Coppersmith, Physical Review B **75**, 115318 (2007).

[11] P. Bas, A. Bostel, B. Deconihout, and D. Blavette, Applied Surface Science **87-88**, 298 (1995), proceedings of the 41st International Field Emission Symposium.

[12] N. Rolland, F. Vurpillot, S. Duguay, B. Mazumder, J. S. Speck, and D. Blavette, Microscopy and Microanalysis **23**, 247 (2017).

[13] G. Voronoi, Journal für die reine und angewandte Mathematik (Crelles Journal) **1908**, 97 (1908).

[14] G. Voronoi, Journal für die reine und angewandte Mathematik (Crelles Journal) **1908**, 198 (1908).

[15] D. J. Larson, T. J. Prosa, R. M. Ulfig, B. P. Geiser, and T. F. Kelly, *Local electrode atom probe tomography* (Springer, 2013).

[16] O. Dyck, D. N. Leonard, L. F. Edge, C. A. Jackson, E. J. Pritchett, P. W. Deelman, and J. D. Poplawsky, Advanced Materials Interfaces **4**, 1700622 (2017).

[17] "Gwyddion documentation, chapter 4: One-dimensional roughness parameters," `http://gwyddion.net/documentation/user-guide-en/roughness-iso.html`, accessed: 2021-06-29.

[18] T. B. Boykin, G. Klimeck, M. Friesen, S. N. Coppersmith, P. von Allmen, F. Oyafuso, and S. Lee, Phys. Rev. B **70**, 165325 (2004).

[19] F. Schäffler, Semiconductor Science and Technology **12**, 1515 (1997).

[20] G. Klimeck, S. S. Ahmed, N. Kharche, M. Korkusinski, M. Usman, M. Prada, and T. B. Boykin, IEEE Transactions on Electron Devices **54**, 2090 (2007).

[21] M. Friesen and S. N. Coppersmith, Phys. Rev. B **81**, 115324 (2010).

[22] "Bernoulli Distribution," in *Statistical Distributions* (John Wiley & Sons, Inc., Hoboken, NJ, USA, 2010) pp. 53–54.

[23] S. Aja-Fernández and G. Vegas-Sánchez-Ferrero, *Statistical Analysis of Noise in MRI: Modeling, Filtering and Estimation* (Springer, 2016).
