# Supplementary material for: Atomic fluctuations lifting the energy degeneracy in Si/SiGe quantum dots
Source: Nat Commun. 2022 Dec 13;13:7730. doi: 10.1038/s41467-022-35458-0 (PMC9747794; doi:10.1038/s41467-022-35458-0)
Supplement: Supplementary file 2 — Description of Additional Supplementary Files [file 41467_2022_35458_MOESM2_ESM.docx]

File Name: Supplementary Movie 1

Description: Deviation of each isosurface tile position from the isosurface’s average position for the top interface of quantum well B (for increasing Ge concentration).
